# Becoming InterActive for Life: Mobilizing Relational Knowledge for Physical Educators

**DOI:** 10.3389/fspor.2021.769031

**Published:** 2022-01-12

**Authors:** Rebecca J. Lloyd, Stephen J. Smith

**Affiliations:** ^1^Faculty of Education, University of Ottawa, Ottawa, ON, Canada; ^2^Faculty of Education, Simon Fraser University, Burnaby, BC, Canada

**Keywords:** interactive flow, kinesthetic consciousness, phenomenology, flow, salsa dance, Tai Chi, acroyoga, equestrian arts

## Abstract

The overarching purpose of the InterActive for Life (IA4L) project is to mobilize relational knowledge of partnered movement practices for physical education practitioners. Through a participatory, motion-sensing phenomenological methodology, relational knowledge gleaned from world class experts in salsa dance, equestrian arts, push hands Tai Chi and acroyoga, and analyzed through the Function2Flow conceptual model, was shared with Physical Education Teacher Education (PETE) students. They, in turn, made sense of the ways these experts cultivate relational connections through a process of designing interactive games suitable for physical education curricula. The kinetic, kinesthetic, affective and energetic dynamics of these games were then shared through professional development workshops, mentoring, and open-access resources. Each phase of the IA4L project invites us to depart from the predominance of individualistic ways of conceiving and teaching movement and instead explore what it means to be attuned to the pulse of life as we break away from tendencies to objectify movement as something our bodies do or that is done to them. Consideration is given to the ways in which meaningful relational connections are formed in and through movement and how this learning prioritizes the InterActive Functions, Forms, Feelings and Flows of moving purposefully, playfully and expressively with others. In so doing, what this research offers is an understanding of how knowledge of an essentially motion-sensitive kind, which can breathe life into physical education curricula, can be actively and interactively mobilized.

What useful knowledge for physical education emerges when we research expert practices of partnered physical disciplines? What can be learned from such practices for becoming not just active but also inter-active for life? And what can physical educators make of this relational knowledge that contrasts with the predominance of individualistic ways of conceiving and teaching movement, such as the isolated sport techniques and so-called fundamental movement skills that have a stranglehold on physical education pedagogy worldwide (Kirk, 2010, 2011, 2013, 2019)? What if we departed from Cartesian representations of an objectively rendered body in physical education resources and assessment tools (e.g., Cairney et al., 2018, 2019; Edwards et al., 2018; Stearns et al., 2019) and instead paid attention to the relational dynamics of movement, the felt sensations, and to the feelings of moving with and being moved by another sensing, feeling person?

The InterActive for Life project is based on phenomenological explorations of the “felt sense” (Gendlin, 1962) of moving and being moved in response to an animate other, be it a partner on the dance floor, an opponent in combative play, someone supporting and balancing us gymnastically, or even with a horse when engaged in liberty play. The impulse to move doesn't necessarily arise from individual volition, considered as an essentially cognitive process of decision-making, but more as an “interbodily resonance” (Fuchs and Koch, 2014, p. 6; Fuchs, 2017), an “interkinesthetic affectivity (Behnke, 2008), or as what Shotter (2006) termed a “withness” that deepens through these motion-sensing practices.

What “participatory sense-making” (Fuchs and De Jaegher, 2009; Hermans, 2021) can we draw from these partnered, disciplinary practices as phenomenological researchers? How, in fact, can we best study interactional dynamics phenomenologically when, according to De Jaegher et al. (2017), “practical or empirical phenomenology has so far not focused much on interactive experience” (p. 494)? And in sharing “fine-grained” understandings of relational dynamics (Satne and Roepstorff, 2015, p. 13; Satne, 2021) with physical education teacher education students, what practical findings might emerge that inspire teachers to consider more deeply the ways of cultivating meaningful, relational connections in physical education (PE) pedagogy at large?

These are the questions guiding a 5-year, multiphase SSHRC funded project where relational kinetic-aesthetic-kinaesthetic energetic knowledge, as conceptually framed by the Interactive Function2Flow model^1^, was gleaned from world class experts in partnered activities such as salsa dance, acroyoga, equestrian and martial arts (phase 1), generalized into interactive games and activities that were co-created with Physical Education Teacher Education (PETE) students (phase 2), and shared with emerging and seasoned physical educators through professional development workshops, mentoring, and open-access resources (phase 3). This project design, where various means of knowledge creation and mobilization have been interwoven throughout, puts forth a ‘hands on' rendition of knowledge of a deeply relational, motion-sensing kind.

The particular focus of this article will be on what emerged in phases two and three of this 5-year IA4L project and the meanings PETE students make of prioritizing relational knowledge within their developing practices during a time when they were also experiencing COVID-19 government-mandated restrictions (Vilchez et al., 2021). While we will make reference to phase one data to set up enough context to understand what inspired the various ways of cultivating meaningful connection, our goal is not to repeat the theorization of this phase since it may be accessed elsewhere. For example, phase one data was showcased in publications pertaining to interactive flow in salsa dance, with particular attention given to the responsive act of “following” (Lloyd, 2021), and to the subtle to-and-fro oscillations of “leaning in” when engaged not only in salsa but also in acroyoga, equestrian arts and push hands Tai Chi partner practices (Lloyd and Smith, submitted). The in-depth phenomenological underpinnings of the “motion-sensing methodology” to this research were also published elsewhere (Lloyd and Smith, 2021). They were inspired by Merleau-Ponty's (1962, 1968) way of making sense of the body and broadened to kinaesthetic and affective realms through the “radical phenomenology” of Henry (2008, 2015), the “kinetic-kinaesthetic-affective” analyses of Sheets-Johnstone (2011, 2014, 2017, 2018, 2020), and the intersubjective, affect-oriented processes articulated by Stern (2002, 2004, 2010).

We now direct attention toward making sense of becoming interactive in ways that prioritize the felt sense of life communicated physically through the relational dynamics of posture, stance, position, and expression in PE contexts. Such a focus adds a much-needed layer to PE resources and assessment tools (e.g., Robinson and Randall, 2017; Edwards et al., 2018) that do not take into consideration how to adjust the force, timing and aim of a movement pattern, be it a walk, run, or throw, in keeping with how the person with whom you are moving feels. This focus allows us to create practical and accessible interactivities to help physical educators make meaning of “the motile aspects of the human embodied dimension” (Whitehead, 2004, p. 4), which is a known area of struggle for many physical educators who wrestle with what embodiment actually and practically means (Stolz, 2013; Jurbala, 2015; Standal, 2015; Stoddart and Humbert, 2017; Robinson et al., 2018; Harvey and Pill, 2019). When we take time to discern the very kinetic, kinaesthetic, affective and energetic registers within the activities that comprise the practical subject matter of PE curricula, we can shift our focus from the skillful processes of becoming physically active to the functional, formal, felt, and flowing ways of interacting meaningfully with one another.

## Motion-Sensing Phenomenological Methodology

A phenomenological methodology coupled with interview analysis methods of qualitative research best serve the overarching purpose of this study which is to mobilize knowledge from partnered practices in ways that inspire meaningful relational connections in physical education pedagogy. We draw upon van Manen's (2014, 2016) methodological guidelines for hermeneutic phenomenology and, given that the data gathered in this study pertain to better understanding movement experiences, particularly experiences of flow where interactions are sensed between self, other, and the milieu of action, we have extended van Manen's work in the direction of “motion-sensing phenomenology” (Lloyd and Smith, 2015, 2021). To have this phenomenological methodology be “motion-sensing” means providing vivid, experiential descriptions of moving together with one another that are then subject to existential, theme-based analyses. Such descriptions provide a deeper understanding of the phenomenon of interest in terms of the functions, forms, feelings and flows of interactive *relationality* (Lloyd and Smith, 2006; Lloyd, 2016; Smith and Lloyd, 2020). In so doing, this methodology furthers the phenomenological conceptualization of flow theory (Csikszentmihalyi, 2000, 2008, 2014) and lends itself practically to the professional development of physical education teachers and to the creation of resources that will be of use to physical activity participants at all levels.

### Participants

Phase one of the study features interview and video documentary data of world-renowned teachers of partnered disciplines including the founders of AcroYoga Montreal, Eugene Poku and Jessica Goldberg, Taijiquan master Sam Masich, two-time world Salsa champion, international judge and coach, Anya Katsveman, and Equestrian artist and master horse trainer, Paul Dufresne (as featured on https://function2flow.ca/phase-1-learning-from-experts/). Inclusion criteria for these participants were individuals who: engage in an inter-activity on a regular basis (3–7 times per week); have accrued a minimum of 10 years of experience; have shared their knowledge about this inter-activity with others; and are recognized globally as expert practitioners.

Phase two of the study features two Physical Education Teacher Education (PETE) students, Anika Littlemore and Christina Nyentap, who took a particular interest in mobilizing the relational knowledge gleaned in phase one of the study for physical education teachers. They volunteered to be part of the ethics-approved, professional development-oriented component of the InterActive for Life research project and, over the course of the 2019–2020 academic school year, met on a weekly basis with the first author of this article to experience first-hand the relational postures, positions, gestures and expressions that were initially exemplified in the disciplines of push hands Tai Chi, salsa dance, equestrian arts and acroyoga with the goal of adapting them to PE contexts.

Through playful, collaborative and emergent inquiry, a series of games and relationally-oriented activities, what we frame as ‘interactivities', were developed and co-presented through professional development workshops offered to other students in the Bachelor of Education (B.Ed.) program at the first author's home university as well as to professionals in PE (i.e., the 2019 Healthy School Communities National Forum) and coaching contexts (i.e., the Sport for Life Summit, 2020). They also contributed to the creation of an open-access IA4L resource featuring a series of interactivities that can be used in professional development workshops online or via web-based access and dissemination (Nyetap et al., 2020).

Phase three participants responded to our strategic social media knowledge sharing (Cooper and Shewchuk, 2015) through various social media platforms, including Twitter and Instagram (Freire and Lloyd, 2021), and expressed interest in journaling their experiences of interactive function-form-feeling-flow in their emergent physical education practices while receiving online professional development mentoring. Two PETE students from Brock University, Matt Dingwall and Eliza Herter, opted to reveal their identities on their ethics-approved consent forms and meet on a bi-weekly basis between January and May 2021—a time when they were experiencing a combination of in-person and online practicum placements in physical education. The aim of this mentoring was not simply to help these PETE students translate the knowledge gained from the first two project phases as guidance for their own emerging PE practices but for them to literally take up what resonates with and moves them to become interactive for life and thus to inspire their own students similarly.

In August of 2021, all four participants, with the addition of two graduate students who also signed ethics-approved consent forms, came together for the filming of a mini documentary where they were invited to move through some of the inter-activities comprising the IA4L resource and respond to open-ended prompts asking them to describe the InterActive Function2Flows of what they experienced which they intend taking into their PE teaching practice.

## Mobilizing Knowledge of Partnered Practices

We now share the meanings that our Physical Education Teacher Education (PETE) participants made of the interactive practices that were inspired by what we learned from experts in acroyoga, push hands Tai Chi, salsa dance and equestrian arts and what we incorporated in the interactive curricular resources. Each Interactive Function, Form, Feeling and Flow (IAF2F) section begins with a summary of relevant excerpts taken from phase one interviews with world class experts in partnered disciplines that is followed by examples of PETE students making sense of what they learned interactively. Knowledge mobilization is shown to be not simply a translation of research findings into practical applications but, rather, it can be a process transferring and literally trans-positioning movement-sensing insights in a way that transforms Physical Education practice.

### InterActive Function: Connecting Postures

Each PETE participant was inspired to think about the subtle ways that posture and presence can cultivate feelings of connection. What our experts in partnered practices revealed is that, regardless of the activity discipline, and whether your partner is a horse, dance partner, martial arts opponent or fellow acroyogi, the initial contact is essential to building a sense of connection and rapport. When approaching a young horse, for example, one must assume a calm presence. Expert trainer Paul Dufrense, when interacting with a young horse, explains that “she is going to be terrified of everything in her environment. At this age they don't survive unless they stay really aware” (Aug, 2018). This means that if you are unaware of the extra tension you are carrying in your body or breath, the horse will let you know that by how they respond to you in the initial moment of greeting. If you are too tense, they will want to move away rather than toward you. Similarly, the first sign of greeting a partner in salsa dance is also essential for building a connection. Anya Katsevman, two-time world champion salsa dancer and judge, asks her partner to consider “how my body is to you, whether it's open, what my posture is like. Am I looking at you? Are you looking at me?” (May, 2018). Rather than concerning oneself with the technique of the dance, the first step is to consider how comfortable it is to approach someone in the invitation phase of asking that person to dance. Is there a sense of readiness to form a connection that may be discerned through postural presence?

While PE students might not be connecting with horses or dance partners, many fitness activities, drills, games and sports that form the content of PE programs require that students interact with each other on a regular basis, yet the initial signs of readiness to form a meaningful connection with someone are not necessarily addressed. With the goal of prioritizing the felt sense of connection between participants in PE, PETE students Anika Littlemore and Christina Nyentap developed a series of warm-up activities. In the process of developing these inter-activities, they were inspired to think about the shifts that were happening within themselves in terms of their readiness to connect with others. They spoke of the predominantly individual focus they usually had when they engaged in fitness activities and what emerges when they become more relationally aware. What came up for Christina was the importance of paying attention to the relative signs of comfort as an interactivity begins. It is something she thinks we should also pay attention to when students are paired in PE classes.

I knew Anika from classes. And I was ready to connect with her. We were both part of the IA4Lproject and we were both ready to connect. What I find, however, is that when I did a partner activity with my boyfriend [at home during the COVID-19 lockdown], his first reaction was, like, why are you doing this? Why are you staring at me? He wasn't comfortable at first (Nyentap, March 2021).

Anika had a more synchronous experience when she applied the principles of the IA4Lproject to her own life and at-home workouts with her significant other when the gyms were closed due to the COVID-19 lockdowns.

I used to go to the gym by myself and I'd want to just be in my own little music bubble doing my own thing. But now I much more enjoy doing something together…

When I'm doing squats with him, we're kind of going at the same time, so watching him and seeing his movement reminds me about my own movement, if that makes sense. I don't have a mirror anymore. At the gym I would have a mirror and I would look at myself and kind of connect between what I'm seeing and what I'm feeling, but now I'm looking at him and becoming aware of my micro movements (Littlemore, March 2021).

What Anika was describing is an inner awareness of overall postural presence that emerged from facing and making eye contact in a squat. By seeing how her partner was experiencing the squatting motion she, in turn, was able to feel what she was seeing in her own movements. For such a deepening of awareness to occur, the initial comfort in facing her partner needed to be there. For many students in PE, however, especially now in this COVID-19 pandemic context, we need to consider what it might be like to make those initial moments of connection.

Matt Dingwall and Eliza Herter, PETE students who made sense of their PE practicum placement through the tenets of the IA4Lproject, became aware of the postural signs of readiness for their students to engage in games and sports. They noticed discrepancies between students who were there ready to play and those who seemed to be in their own bubbles. Matt spoke, in particular, of two girls walking up and down the court in a territory game of Tchoukball while engaged in conversation with each other. They never received a pass and, when asked about it on the sidelines as the teams rotated, they were not aware of body signals that would communicate to teammates that they were ready to interact. Matt recalls:

I was trying to get them engaged, not just with each other, but with other students as well. So I started getting a little animated and demonstrated what the ready position looks like. By the next time they were on the court, they weren't doing exactly what I was doing but they were starting to engage. Getting these two girls involved meant more to me than them learning any skill. They initially had no interest in being in that class, yet they actually started playing a little bit. That was like a big win for me and at that point I didn't really care if they caught a ball (Dingwall, February 2021).

He further reflected that it seemed like no one ever took the time to teach them how to enter into the game and show that they wanted to engage with their teammates. He critically compared the importance of teaching these signs of physical readiness to the disconnected ways fundamental movement skills are taught in isolation thereby missing this communicative dimension entirely.

Eliza Herter, a PETE student who also participated in this online interview, noticed similar signs of postural disconnection in a badminton game she observed during the COVID-19 outbreak.

Most of the class members, if I'm being honest, don't really speak to each other, which again was weird. There are only six students in the class. They don't come into class every day either since they're on a rotating schedule. One boy was very much alone and just there, but not really making any eye contact with the others. And then there was one kid who was super into it and he's bouncing on his toes (Herter, February 2021).

With concern for recognizing and responding to students who may not be ready to connect or lack a taken-for-granted-knowledge of what it means to communicate signs of wanting to connect with others, the IA4L team consolidated the relational knowledge inspired from experts in partnered practices and the reflections of our PETE participants and formed a series of inquiry-based prompts. The following questions, drawn from the Function2Flow phenomenological analysis, serve as a guideline for PE teachers to consider before a game or activity begins:

*What does a ‘ready to interact' posture look like? Is there an optimal alignment, tone and/or tension? Where is your weight distributed to create connection? Is it forward/ in the centre of your foot/ back into your heels? What relationship does your postural alignment have to the floor? Is it low and connected to the ground? Is it lifted from a certain place? Is it grounded in a certain place? In what ways can you adjust your posture to better connect with your partner?* (InterActive Function2Flow Model, 2020^1^).

If students show noticeable signs of discomfort in making an initial connection, such as demonstrating closed positions or withdrawal in the eyes, teachers may want to offer alternatives, such as shifting a partner throw-and-catch activity to interacting with an inanimate surface such as throwing a ball against a wall. And if the beginning stages of readiness to connect are observed, a teacher then may want to introduce an interactivity such as Rock-Paper-Scissors-Glue to build a baseline sense of connection where students are invited to face a partner and eventually move together in synchronicity. To teach toward a sufficient level of InterActive Function means learning to develop the capacity for kinetic connection that can be felt in a variety of simple mimicking and matching motions.

### InterActive Form: Connecting Positions

The partnered activities studied in phase one of the IA4L Project ranged from up-close, body-melding interactions of acroyoga to incrementally distanced partner dynamics of push-hands Tai Chi, salsa dance and equestrian arts. Despite any increase in distance, connection is maintained via postural and positional cues. For example, in salsa dance the connection is experienced not only through the interconnected palms but also via the forward-angled lean through one's entire bodily posture and position. Two-time world salsa champion Anya Katsevman explains:

Because we are communicating with each other, it's important to give each other weight. Connection happens through body weight; therefore, as part of our normal posture, we have to take the entire bone structure of the body which usually is controlled by the pelvis—because the pelvis will move the spine as a whole—and put it toward the balls of the feet as opposed to the middle or the back so that our weight can be closer to each other. When both of us maintain that, we can always feel connected without creating any extra force or pressure. I think this is applicable everywhere. You want to touch someone, you press on them, right? So my weight will always be forward toward my partner and that allows us to be connected all the time (Katsevman, March 2018).

With the intention of mobilizing this sense of postural and positional connection for PE, the PETE students and IA4L researchers co-created the game Leaning in Mirror Walk during their weekly movement inquiries over the 2019–2020 academic year. At first partners were invited to face each other and maintain the distance between them while a nominated leader in the partnership led the follower in a multi-directional mirrored walk. What inevitably transpired was an inclination to lean back and away from one's partner when it was time for the follower to move backwards. To mitigate this flight or startle impulse to physically retreat or disengage, which somatic practitioners refer to as a “red light reflex” (Hanna, 1988), partners were invited to press their torsos against an exercise ball or hoop held between them. If either partner broke free from the forward angled connection, the ball or hoop would drop to the ground and give them instantaneous feedback that their form had changed. What became apparent is that this positional sense of multi-directional connection is applicable to a wide-range of sports, such as the offensive and defensive notion of shadowing or matching an opponent while traversing a playing field, rink or court.

When Matt Dingwall from Brock University recently traveled to Ottawa to be featured in a documentary video capturing our PETE student experiences with the IA4L project, the Leaning in Mirror Walk was the activity that stood out for him more than any other. Despite his prolific experience in hockey and other competitive sports, he found it incredibly challenging to maintain a forward-angled presence when moving backward. He said:

During the beginning of the resist-a-ball activity, I felt a sense of disconnect. At times I felt like my joints were not fluidly moving, and I felt that there was a feeling of static movement in my body. As I fed off you, my partner in the activity, I started to feel a sense of connection in how you were leading that allowed me to lean in and immerse myself in the activity and feel a sense of flow. This was exemplified further when the two of us closed our eyes and were able to move fluently together without the ball dropping (Dingwall, August 2021).

Matt's insight is that engaging in partnered activities can foster other-to-self, relational pathways that draw attention to bodily form, feeling and flow. As described more fully in Lloyd and Smith (submitted), the core-to-extremity awareness cultivated in the partnered balances of acroyoga, supporting the point of contact in push-hands Tai Chi, and developing an intuitive sense of when a follower in salsa should angle themselves forward toward their leader (Lloyd, 2021), indicates that the more one engages in such partnered practice the more one can develop the relationally-oriented body awareness necessary for sustained interaction.

This body awareness takes practice in developing the ability to read and attune ourselves to the bodily positions of others. When Paul Dufrense cues his horses to move in circular and figure-of-eight patterns, for example, he pays close attention to the amount of bend he observes in the horses' torsos and the degree to which their heads turn back toward him. He describes working with a mare in a round pen.

As she runs a smaller circle, she causes herself to bend around me. The more she does that, the better she will be emotionally. When the horse makes that outside turn, she should come around and look for me. And when she does that, she creates bend in herself. Now that becomes a positive. I actually cause a compression in that horse for her to soften (Dufrense, August 2018).

PETE student, Anika Littlemore, spoke about how this positional awareness described in equestrian interactivity inspired her to think of her bodily presence in soccer differently.

When Paul, the equestrian, was saying how he was connecting with the horses through space, it's like soccer which, for me, is connecting to my teammates in the space that we play in. I never really thought of it before. I was just in the space and the interaction was just happening. But then I started really seeing what's going on between Paul and his horse and how subtle movements can deeply affect the connection between him and that animal (Littlemore, March 2020).

Anika then described the importance of seeing where her teammates' shoulders, hips and feet were angling in developing a sense of where they will send the ball in a soccer game. She described the importance of relative positions and the meaning they have for developing a bodily language of communication.

Am I turning myself this way? Am I facing the center of the field? Am I facing a certain person because I am wanting the ball from them? Or if I am forward, if I'm attacking and if I am wanting to get a ball from one of my teammates, then I'm coming into the space, I'm coming toward them and facing them. Shoulders square to someone means that I want the ball on my feet. If my shoulders are facing forward and not toward my teammate, or if they face sideways, I want the ball in the space so that I can run onto it. When you're pointing your shoulder, you're also pointing your feet. So when you have a split stance, my front foot is telling you I want the ball here. Or I want the ball on this foot so that I can touch it one time and then run. The cues are subtle. But also, if there are people you haven't played with then you're going to tell them by pointing to where you want the ball to go (Littlemore, March 2020).

PETE student, Christina Nyentap, elaborated further on the gestures and relative positions that are part of an interactive tactical awareness in offensive and defensive play. She referred to it as an inner awareness that she said she never learned in her PE classes when growing up because she always had her focus directed outward to the ball.

I think it's always the outward focus. So, let's say we're doing a basketball drill. And we're learning to defend against another player. I need to make sure that I stay with them. But it was never inward. It was focusing on the ball because I don't want them to score. We were not taught to focus on gestures and eye contact (Nyentap, March 2021).

Thinking beyond the ball to the visible actions and reactions communicated by teammates that inform one of where to send the ball is, as Christina points out, not something she was taught to pay attention to in her PE classes when she was a student. To help mobilize this knowledge in the PE context, the IA4L research team developed the game Geometric Kickball which incorporates communicative gestures. When the game is played initially, the person receiving the ball points to the location on an identified shape marked on the ground with chalk (or with pilons) where they would like to receive the ball. As the game complexifies, the receiver gradually communicates more discrete signals such as head, shoulder, torso, hip, and foot angled cues. This, as well as other interactivities that Anika helped to co-create such as Mirror Cone Touch, are designed to help learners communicate through their relative positional cues.

To help mobilize such positional knowledge beyond these games, a series of inquiry-based prompts were developed based on the Function2Flow phenomenological analysis. Here we ask PE teachers to consider the following when introducing games and activities:

*What ways can you position yourself through space to improve interaction? Describe the torso twists, angles, and turns of optimal interaction. Are you able to sense how twists and turns of your shoulders/torso/ hips/waist create connection? Are you able to sense how leaning more towards your partner or away affects feelings of connection? Are you aware of how changing your relative level, i.e., crouching down or standing up taller affects feeling of connection?* (InterActive Function2Flow Model, 2020^1^).

### InterActive Feeling: Connecting Sensations of Timing and Force

Each expert in the partnered disciplines spoke about the kinaesthetic feelings that emerged as they were silently interacting and communicating. No matter if the connection be felt through greater distances when engaging with horses, or if it be the compression of intertwined palms in salsa dance, or the full-body suspension of a flier by a base in acroyoga, the feelings of connection are inevitably experienced in reciprocated, wave-like calls and responses. Tai Chi Master, Sam Masich, relates this kinaesthetic interactive responsiveness to what is experienced in contact dance.

You can think of perceptual movement in terms of contact improvisation. When I take a step and I'm in this position, my arm naturally counterbalances the step….my entire body knows how to move based on that open, fascial connection. This could be called the listening bell—so like with the normal bell that if you touch it anywhere the whole bell rings. What we want to do is create conditions where, if the partner touches us here, or pulls us here, or grabs us here, or punches us here, no matter what they do, the whole body becomes connected to that action. The training is to make this response more and more efficient and more and more whole body. So it's not some motion toward me that I bat away like a reflex in a fight or flight response. Instead I need to connect with it. I need to listen to the fascial bell. When we do that we get strange and productive martial arts results (Masich, October 2018).

This counter-balanced, active and interactive, dance of kinaesthetic responsiveness was also described by Jessie, the female in the acroyoga partnership who assumes the role of the base. When supporting her husband Eugene in balanced, airborne yoga poses and transitions, there is no time for verbal feedback or conversation. A sense of knowing how to respond in a supportive way is cultivated through the reciprocating motions of the practice itself. Jessie Goldberg explains:

The base's primary responsibility is to stay underneath the flier. If we are doing a seated, L-shape position, my job is to stay underneath. So if my flier is too far back or too whatever, there is no time for me to correct it verbally. I just get under there and then I bring it back (Goldberg, December 2019).

Such a connection is not one-sided. Eugene describes what it is like to experience a supported balance with his partner vs. experiencing a press on an inanimate surface such as a sidewalk. He asks us to imagine how our walking would change if the surface upon which we placed our feet became alive and started to participate in the experience—a phenomenon that exemplifies a Merleau-Pontian (1968) chiasmic intertwining which adds an extra degree of liveliness that has been previously ascribed to walking (e.g., Ingold, 2004; Midgelow, 2018).

When I'm with Jessie, it's like I'm on the ground. Jesse is the ground and she is soft. She is a cushion... like a sidewalk which has a little give. So, that's the first thing. The second thing is how I react to the ground. I could just stay here and then you can start manifesting, or I could push back and we could manifest together. That's the reciprocity, in every way. So imagine every time we took a step, the ground was thinking. Every time I touch the ground, the ground thinks (Poku, December 2019).

We can connect this kinaesthetic responsiveness to the ways we might enhance connection with others in PE contexts. Anika and Christina experimented with partner-based fitness activities, such as the stationary partner squats and handstands that are featured in the game Lean on Me created by the IA4L team as well as the locomotor Walk the Line interactivity which mobilized these relational pressure sensations into locomotor actions and reactions. Yet developing an inner kinaesthetic awareness in and through partnered interactivity takes time for PETE students. When asked about their experiences of interactive kinaesthetic connection, both Anika and Christina referred to the process of learning to refine a partner handstand by sensing the pressure communicated through their feet. Anika explains:

For me, the eye contact is kind of like the base. This is how I'm focused. And then I start to feel where we're connected. Our feet are connected so then you feel the feet. That's where I can feel either her pushing in on me, or me pushing on her. Are we pushing on each other equally? Is my foot pushing outwards? Is her foot pushing? Do I need to push inwards? That's what I am feeling. Then I also feel my core engagement. Am I engaging my core? Am I stable? Am I shaking? Am I the one who is starting to wobble? There are just those subtle cues of trying to figure out where I am and where she is and where we both need to move (Littlemore, March 2020).

Christina adds to Anika's description:

If I push too much on her then I'm going to push her over. Okay, if she pushes too much on me then she's going to push me over. So we have to create a balance where it's a helpful amount of force, not a hindrance because, like I said, you can lose balance and push each other over or you just won't be successful. We won't get up in the air because you know one person's pushing too much on the other. So when Anika would push more on me, then that would mean I'd have to push back more on her for us not to topple over. You have to almost have equal amounts of force, even though we don't weigh the same. We would balance that weight difference by pushing, allowing equal amounts of force against each other. And then, in terms of going up, we both knew the goal but sometimes we fell over probably ten or more times before we got up. But eventually, as long as we were both concentrating and listening—and based on the force and the direction of where we wanted to go—we would get up (Nyentap, March 2020).

What stands out in the above passage when Christina describes a language of communication mediated through pressure is her reference to listening. To listen is to sense the sound qualities of a movement. To feel is to drop kinesthetically into what we hear such as the resonating sound waves of a bell. To help educators move beyond generic recommendations to “listen to your body” and move toward the kinaesthetic realm, the IA4L team developed a series of InterActive Feeling prompts from the phenomenological analysis that orient us to sensing the body language mediated through timing and pressure:

*What ways can you adjust the timing and force of your macro movements and subtle gestures to get a better feeling of connection? What ways can the timing and force of gestures influence the sense of connection? Can you feel the impact of quick/slow, heavy/light, or sustained/choppy actions? What helps you move beyond the bounds of predictable rhythm and embrace moments of surprise?* (InterActive Function2Flow Model, 2020^1^).

### InterActive Flow: Connecting Energies

When experts of partnered practices move in kinaesthetic, interactive response to sensations of force, positive sensations emerge, not only in mood, but synergistically in the ease of the motions themselves. Eugene, the flier in the expert acroyoga partnership, is very much aware of “the things I do in my body, or in my foundation, to be able to receive the energy that's been given to me and also give energy back” (Poku, December 2019). When such reciprocity emerges “there is an ease in the movement. There is an ease in the breath. There is an ease in the communication. It's a flow, a natural flow, just like breathing” (Poku, December 2019).

Anya Katsevman, describes what it is like to experience a similarly interactive exchange in salsa dance:

I think meditation is the closest thing to this kind of practice. Or any kind of listening. When I'm a follower, I could just follow (laughs) and see what he wants because potentially he wants to do something I don't know exists. So I get out of my head because what he wants isn't there. This makes it more fun for me. But if there's a perception in my head of what should happen, then I deprive myself of the spontaneous experience (Katsevman, December 2018).

When this level of openness exists between the partners, as equestrian artist and trainer Paul Dufrense describes, a synergy between the lead and follow exists.

The horse is going to allow you to make changes to how they move, or where they go, with no apprehension. They'll think, “Oh, this way over here.” And they'll go there. What will happen is that you are using a minimum of energy to create it (Dufrense, August 2018).

Unlike the decision-making focus and cognitive underpinning to many physical education models and teaching styles (e.g., Bunker and Thorpe, 1986; Goldberger et al., 2012; Casey and MacPhail, 2018) interactive flow emerges when one lets go of any preformed decisions or plans. Paul Dufrense further explains:

This is going to be more like freestyling. I have an idea and let's see what we get, as opposed to running a set pattern. When we run a set pattern we suck the life out of it, rather than encouraging interaction in a much more delightful way. It becomes a lot more fun. All of a sudden everything picks up this life (Dufrense, August 2018).

It is important to note such openness to move in spontaneously interactive ways takes time to develop. It does not happen right away. A level of trust must be earned. Tai Chi master Sam Masich describes such responsiveness as a cultivation of instinct:

what I'm learning to do is, through my connection with the other person, work from my natural instincts toward more cultivated responses. My connection with a partner takes me to a place where I am responding better. I am responding by saying “yes” rather than by saying “no” all the time. It's not a fear-based response but a connection-based response, although it's not easy to get there in anything we do in life. Push hands practices and the rules that support push hands practice are designed to take us in this direction and ultimately make us better people (Masich, October 2018).

Mobilizing this knowledge of interactive flow in physical education contexts requires consideration of many preparatory factors. PETE student Christina Nyentap points out that, first, when we are grouping students to engage in any sort of partnered activity, we need to be aware of their apprehensions and fear-based responses.

I think kids might feel awkward, even in high school, when they would do a dance unit. We tried ballroom dancing on practicum. There were a couple times when we had a guest person come in. And everyone was, like, “I don't want to touch my partner.” So if there is apprehension, the students are just not ready. They have to be able to get into the zone. They have to be ready to let go of other worries and just be in the moment. And I don't know if they'd be ready for that… it would have to be something in which they are already well practiced (Nyentap, March 2021).

Christina's concluding thought, that developing a relational kinaesthetic sense would have to be practiced, invites us to rethink the approach we might take toward curriculum planning and ensure that ample time is set aside to purposefully build trust and feelings of connection between students before a unit such as ballroom dance is introduced.

To orient students to the cumulative experience of interactive flow, the IA4L team developed a series of lead-follow games that may be experienced in-person or virtually, such as Guess the Leader, with the aim of helping PE students develop comfort in moving with someone and, over time and with practice, to be able to experience the improvisational flow of interaction. To play the Guess the Leader game, a nominated “detective” or team of “detectives” are invited to discern the secret leader from the follower or group of followers who are mirroring back their every move. It can be done with or without music and in sitting or standing positions or in level-changing locomotion. The key for synchronicity and interactive flow to emerge is a commitment to the practice itself. At first, the secret leader might begin in a self-conscious way worried about what movement to lead next. This is very apparent when there are sharp changes between movement patterns which make it easier for the nominated detective to call them out. But when a sense of connection develops, not only between the players but in terms of the subtle transitions in force, rhythm and amplitude from one movement to the next, it gets much harder to discern the leader from the followers. They are interacting in a synchronously flowing manner.

To play an interactive game such as Guess the Leader is much different than following a pre-set routine or dance/fitness video. There is an emergent, spontaneous quality in the improvisational interaction, where the leader is very much moving in response to affirming “YES” cues of mirroring and matching responsiveness from the followers. Such impromptu action and responsive reactions invite us to consider the possibilities for introducing dance and fitness activities in PE in a way that is relationally attuned to the feelings of the those with whom we are moving. To help teachers cultivate, recognize and teach toward the experience of InterActive Flow, the following prompts from the phenomenological analysis were created:

*What feelings are communicated through posture, position and gesture when you experience optimal connection? Are you aware of your movement expression? Does the quality of your movement signal a readiness to connect? What body parts do you focus on to better connect through movement expression?, i.e., direction and intensity of eyes, openness of the chest. What ways can the timing and force of movement expression influence the sense of connection? Does the sustained intensity of a look or the timing of a smile influence the mood? What feelings of energy/ power/ life emerge in relational flow? Describe the qualitative sensations of optimal movement connection* (IAF2F Model, 2020).

## Conclusion

While much relational knowledge was gleaned in phase one of the IA4Lproject from experts in partnered dance, martial arts, equestrian arts, and acroyoga, it might just as well have stopped there, contained with the disciplines themselves, had we not engaged with PETE students in the second and third phases of the project design. We are delighted by the range of fitness, dance, cooperative and competitive interactivities that emerged over the course of the past year that our PETE students co-created and published (Nyetap et al., 2020; Freire and Lloyd, 2021). We can well imagine the possibilities of introducing students in PE classes to the subtle cueing of relational postures, positions, gestures, and expressions as they, too, cultivate connections with one another in and through the actions and interactions of games, sports, gymnastics, dance, meditative and martial arts, and a variety of outdoor recreations.

To facilitate the process of cultivating motile connections through attention to the interactive functions, forms, feelings and flows, we suggest that the series of prompts we have indicated in each Interactive Function, Form, Feeling and Flow section of this article be adapted to an even wider range of games and sports. We trust this framework will also inspire physical educators to mobilize the knowledge of becoming interactive for life for the sake of adding to the cognitive and behavioral underpinnings of social-emotional learning (SEL) (i.e., Taylor et al., 2017; Corcoran et al., 2018; Eklund et al., 2018) to which curriculum documents ascribe and to affective-oriented learning outcomes (e.g., British Columbia Ministry of Education, 2016; Ontario Ministry of Education, 2019; newLearn Alberta, 2021). We also hope that the IAF2F model provides an accessible and practical framework for dissolving false divides between knowledge, movement skills, and affect-oriented attitudes that seem to be becoming more pronounced in PE assessment at large (Whitehead, 2010, 2019; Robinson and Randall, 2017).

There are no clear divisions between thought, feeling and action when moving animatedly with others. How we come to know and express ourselves is not just an action but an interaction (Sheets-Johnstone, 2014, 2017, 2018) and acting responsibly for oneself and one's wellness is inevitably a kind of “moving in concert with others” (Sheets-Johnstone, 2014, p. 260). What this IA4L project emphasizes further is that thoughtful action and life-sustaining interaction arises ultimately out of kinesthetic and energetic connections with animate others, be they humans or horses. Teaching toward interactive flow means that we depart from any individual conception of what we think we should do to embrace essentially improvisational relationality that is cultivated through interactive functions, forms, feelings, and flows.

PE researchers and teachers alike can take up the process of mobilizing relational knowledge through the motion-sensing means (Lloyd and Smith, 2021) we have described in this article. We hope that this approach to communicating research findings, in our case what we learned from disciplinary experts in partnered practices, inspires other PE researchers to develop their own practice-based understandings so that embodied or kinaesthetic approaches to teaching and learning lose some of their reported mystery and ineffability (Jurbala, 2015; Stoddart and Humbert, 2017; Robinson et al., 2018; Harvey and Pill, 2019). When we think about knowledge mobilization, literally and pragmatically, research findings become less facts to impart and more an active, enactive, interactive process of inspiring others to move. We hope that what we have discussed of our IA4L project inspires other PE researchers and teachers to breathe renewed life and motional sensitivity into the relational practices that comprise school PE curricula.

## Data Availability Statement

The raw data supporting the conclusions of this article will be made available by the authors, without undue reservation.

## Ethics Statement

The studies involving human participants were reviewed and approved by Office of Research Ethics and Integrity. The patients/participants provided their written informed consent to participate in this study.

## Author Contributions

RL conducted the knowledge mobilization activities with the physical education teacher education students that are featured in this knowledge mobilization article. SS assisted with the conceptual framing of writing this article as well as the equestrian and Tai Chi data collection process associated with phase one of the study. All authors contributed to the article and approved the submitted version.

## Funding

This work was supported by the Social Sciences and Humanities Research Council of Canada under Grant number 435-2017-0123.

## Conflict of Interest

The authors declare that the research was conducted in the absence of any commercial or financial relationships that could be construed as a potential conflict of interest.

## Publisher's Note

All claims expressed in this article are solely those of the authors and do not necessarily represent those of their affiliated organizations, or those of the publisher, the editors and the reviewers. Any product that may be evaluated in this article, or claim that may be made by its manufacturer, is not guaranteed or endorsed by the publisher.

## Footnotes

^1^Available online at: https://function2flow.ca/interactive-function2flow-model/.
